# Nanoarchitectonics of a Skin-Adhesive Hydrogel Based on the Gelatin Resuscitation Fluid *Gelatinol*^®^

**DOI:** 10.3390/gels9040330

**Published:** 2023-04-13

**Authors:** Konstantin Osetrov, Mayya Uspenskaya, Faliya Zaripova, Roman Olekhnovich

**Affiliations:** Bioengineering Institute, ITMO University, 197101 Saint-Petersburg, Russia

**Keywords:** gelatin, tannin, adhesive, plasma-substituting solution, biomaterials, antibacterial properties

## Abstract

Hydrogel materials are one of the most versatile representatives of biomaterials. Their widespread use in medical practice is due to their similarity to native biostructures regarding relevant properties. This article discusses the synthesis of hydrogels based on a plasma-substituting *Gelatinol*^®^ solution and modified tannin, carried out by direct mixing of the two solutions and brief heating. This approach makes it possible to obtain materials based on precursors that are safe for humans, while having antibacterial activity and high adhesion to human skin. Thanks to the synthesis scheme used, it is possible to obtain hydrogels with a complex shape before use, which is relevant in cases where industrial hydrogels do not fully satisfy the end use regarding their form factor. Using IR spectroscopy and thermal analysis, the distinctive aspects of mesh formation were shown in comparison with the hydrogels based on ordinary gelatin. A number of application properties, such as the physical and mechanical characteristics, permeability to oxygen/moisture, and antibacterial effect, were also considered. The sorption parameters of the material were characterized in a set of physiological buffers (pH 2–9) using Fick’s first law and a pseudo-second order equation. The adhesive shear strength was determined in a model system. The synthesized hydrogels showed potential for the further development of materials based on plasma-substituting solutions.

## 1. Introduction

Hydrogels are three-dimensional network structures that able to imbibe large amounts of water [1]. They have great practical applicability in medicine. Packaging materials, drug delivery systems [2], prosthetics [3], wound coatings [4], and much more are made on their basis. Despite the extensive range of hydrogel materials developed, their application in practice often does not fully achieve the desired properties. In this regard, the question arises of creating a temporary wound coating with all the characteristics required [5]. Such characteristics include the ability for sorption exudate, vapor and oxygen permeability, adhesion to human tissues, sufficient mechanical strength, and preservation of an antiseptic environment [6].

One task is to obtain hydrogels with a complex shape. This is needed for some types of burn injury [7]. While the industrially produced coatings are not always able to satisfy the user [8], two-component systems that form a hydrogel directly at the application site may have a potential toxic effect [9]. Thus, the specifics of using hydrogels require the development of special materials for each task.

The authors proposed an intermediate option, the synthesis of promising wound coatings based on gelatin and tannin immediately before use, to solve this problem. Such materials have an extensive set of properties required for practical use. Gelatin has a similar structure to collagen, due to which the recipient does not experience rejection upon contact with it. Tannin provides antibacterial resistance to a high number of bacteria. Their combination by means of a simple synthesis (injection molding method) makes it possible to obtain materials with pH sensitivity, adhesion to cells, and the ability to self-repair, depending on the synthesis scheme and the goals of the researcher [10].

**Figure 1:**
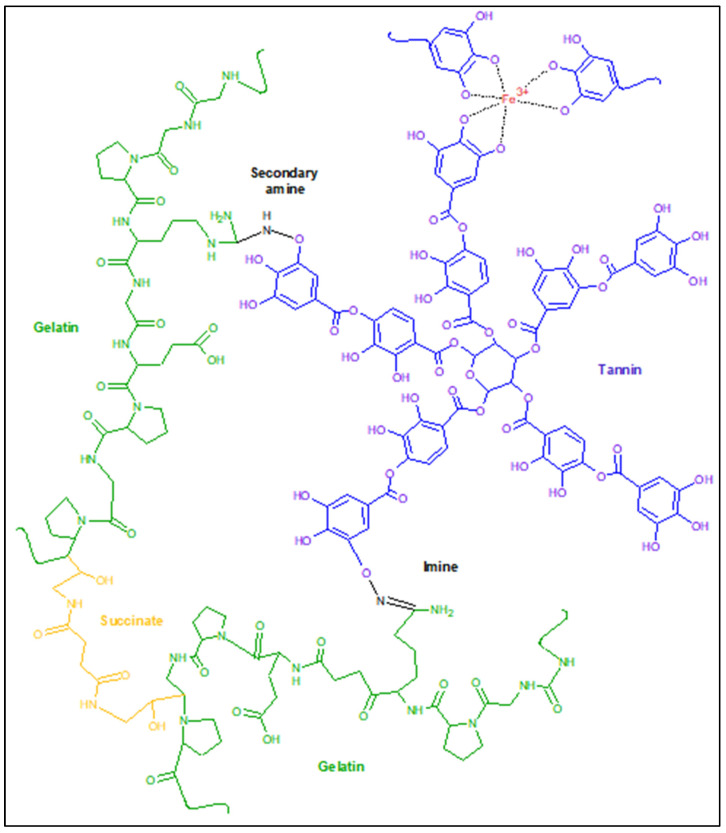
The proposed scheme for the formation of gelatin–tannin hydrogels based on *Gelatinol*^®^.

Gelatin is proposed for use in the form of a succinated gelatin solution with an 8% mass content, which forms a hydrogel after mixing with tannin structured with iron ions and a brief heating (Figure 1). The advantages of using such a scheme are obvious. Among them is the ability to synthesize hydrogel materials with a complex shape immediately before use, but not on the recipient’s body. For example, in cases where it is undesirable to overlap undamaged areas of the human body [11] or where hydrogels of a certain shape are required and where the available coatings do not have the required form factor. *Gelatinol*^®^-based hydrogels can be manufactured immediately before use and with any geometry, using the appropriate shape for molding.

Other medical products of succinated gelatin are also known, such as the blood-substituting solutions *Volutenz*^®^, *Gelofusine*^®^, etc. The main difference consists in the resulting molecular weight distribution, as a result of the use of various types of gelatin, preparation of the monomer, or the synthesis process of the gelatin-succinated solution [12]. In addition, the concentration of gelatin in the above solutions is about 4% mass. After appropriate adjustment of the formulation, it is possible to use them for the synthesis of gelatin–tannin hydrogels, according to the proposed scheme. In the future, it will be possible to carry out synthesis at room temperature by simply mixing the two components after determination of an appropriate catalyzing system. The use of plasma-substituting solutions based on gelatin in combination with modified tannin makes it possible to obtain promising wound coatings with the set of required properties regulated during synthesis. In addition, the use of gelatin-containing solutions has been proven in long-term medical practice [13] and allows us to speak with confidence about the potential safety of the monomer used in this synthesis.

## 2. Results and Discussion

### 2.1. Adhesion Properties

**Figure 2:**
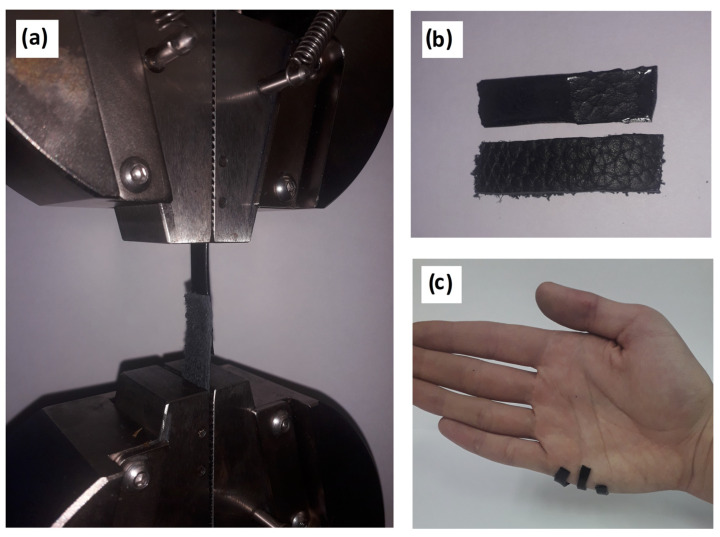
Adhesion experiments: (**a**) installation of a sample for shear testing, (**b**) the mixed nature of adhesive failure, and (**c**) visual demonstration of hydrogel adhesion to human skin.

After drying, the hydrogels showed a sufficiently high bond strength with skin (3.8 ± 0.3 MPa), which was apparently a consequence of the penetration effect. This effect was caused due to the similarity of the hydrogel structure (gelatin mesh) to the pores of skin (collagen nature). It is also worth noting the mixed nature of the destruction of the adhesive compound observed in most cases (70%) (Figure 2b). The remainder (30%) had cohesive bond failure. Significant binding may be a factor in the developed system of hydrogen bonds between the substrate and adhesive. It was mainly bonded through groups of amino acid residues –C=O, –NH_2_, –N= [14]. Equally, a significant contribution was made by the cation-π interaction between the catechol-like tannin residues and substrate structure. A graph of the adhesion bond failure can be found in the Supplementary Materials. The synthesized hydrogels showed an ability for usage as high-adhesive biomaterials, which are in demand for cases of contact with the soft tissues of the body. This includes common medical, wound-healing, orthopedic, dental, and prothesis applications.

**Figure 3:**
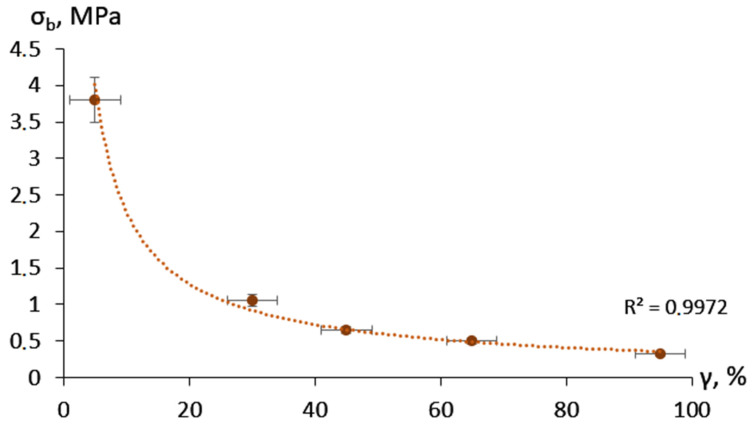
Adhesion bond strength depending on the moisture content.

The adhesion strength showed an exponential decrease with the increase of moisture content (Figure 3). The prevalent mixed nature of the adhesive destruction in the case of the dry material changed to fully cohesive failure above a moisture content of 30 ± 5%.

### 2.2. Mechanical Properties

The tensile strength of the *HP-Fe* hydrogels (γ = 50 ± 5%) reached 80 ± 4 kPa, with a relative elongation at break of 110 ± 5%, which demonstrated the applicability of the materials for use as wound dressings. It is worth noting that *H-Fe* has a lower strength (64 ± 3 kPa) and greater elasticity (150 ± 5%), which sharply contrasts with the results when comparing gelatin films only crosslinked with physical bonds and its succinated analog [15]. Thus, the *HP-Fe* showed a more cross-linked structure compared to *H-Fe*.

### 2.3. Sorption

**Figure 4:**
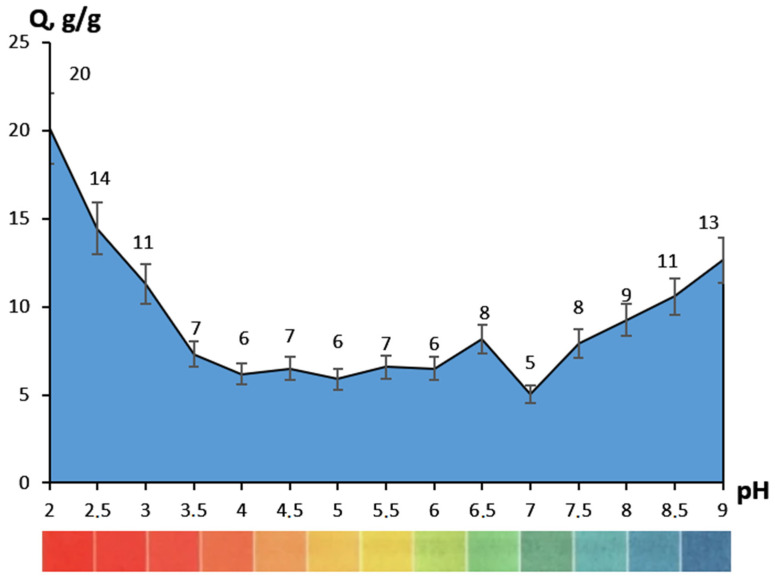
Sorption capacity regarding to the pH of buffer saline.

The hydrogels showed an increased sorption capacity (Figure 4) in acidic (pH < 3.5) and alkaline solutions (pH > 8), which was a consequence of gelatin carboxyl and amino groups ionization, respectively [16]. This pH dependence may have been caused not only by the ability of certain polymer groups to bind water, but also by the relaxation of the mesh and a change in the conformation state of the gelatin molecules [17]. It is also worth noting the discoloration of the materials at pH 2–3. This was observed during the time interval from 1 to 3 h, with an increase in acidity of the solution. This was an indicator of the bond destruction between the iron and tannin ions during the transition from a tris-iron complex to a mono-iron complex [18]. At the same time, the swelling of samples fluctuated within a fairly small range, from 5 to 8 g/g in the pH range of 3.5–7.5. This sharply distinguished them from materials obtained on the basis of unmodified gelatin [19]. In terms of sorption capacity, such hydrogels can be used where pH sensitivity is required; for example, in drug delivery systems. The usage of the proposed hydrogels in the field of wound dressings would require further filler addition, to regulate the sorption capacity.

The samples showed accelerated swelling initially (*k*), with a significant decrease in rate by the end of the study (*k*_2_), according to the sorption velocity (Table 1). In addition, the initial sorption period clearly demonstrated a difference in material behavior in different pH media. The shift of medium to more acidic (pH < 6) or alkaline (pH > 7.5) not only increased the sorption rate 2–10 times, but also changed the nature of diffusion (*n*). The samples ceased to follow Fick’s law (*n* > 0.5), so polymer mesh relaxation prevailed over diffusion. This may have been an indicator of the ionization of the corresponding groups; on the one hand preferring to become negatively charged –OH, –COOH, and on the other positively charged –NH_2_.

### 2.4. Permeability

The hydrogels demonstrated high permeability to both water steam and air oxygen (Table 2). The transmittance rate is affected by a combination of thermodynamic and transport factors. They include the pore size of a material, the mesh parameters of the penetrating substance, and structural features [20]. It seems that the strong affinity of the precursors used for synthesis (gelatin and tannin) here affected the permeability of water. On the other hand, the widely known low crystallinity of the precursors may have been a factor in the high-transparency for oxygen.

### 2.5. IR Spectroscopy

**Figure 5:**
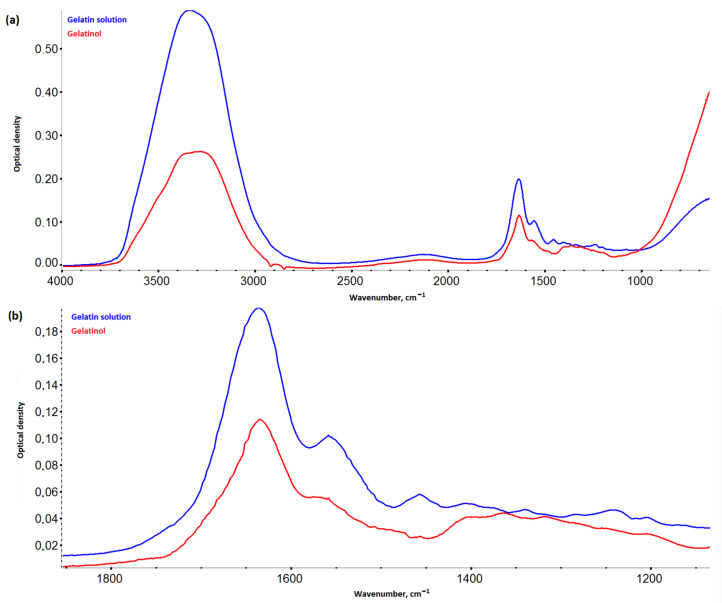
IR spectra of *Gelatinol*^®^ and 8% gelatin solution: (**a**) in the range 4000–800 cm^−1^; (**b**) in the range 1900–1100 cm^−1^.

*Gelatinol*^®^ demonstrated two new peaks, with centers about 1360 cm^−1^ and 1320 cm^−1^ (Figure 5), in contrast to the gelatin solution. These bands were attributed to succinated proteins [21,22], as the appearance of new asymmetric and symmetrical oscillations of the carboxyl group. A significant decrease in intensity over the entire spectrum was probably due to the formation of more massive and more compact molecules bound by gelatin succinate residues.

**Figure 6:**
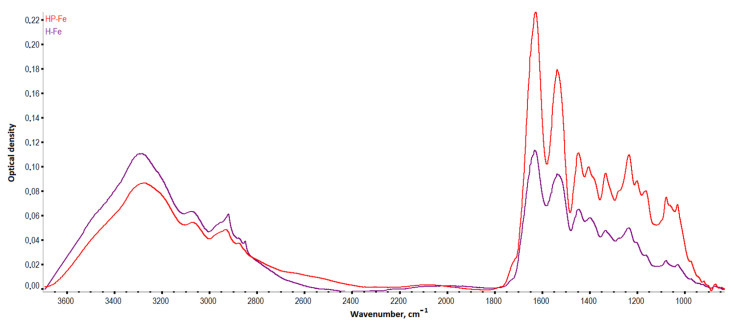
IR spectra of the hydrogels.

There was also a noticeable decrease in the intensity of vibrations in the region 3700–1300 cm^−1^ and an increase in intensity in the region 1200–1000 cm^−1^, when comparing the spectra of the hydrogels (Figure 6) based on *Gelatinol*^®^ and gelatin. An increase in the intensity of peaks with centers around 1165, 1080, 1030, and 973 cm^−1^ (planar deformation vibrations of the C-H bonds of benzene rings) indicated a greater contribution of tannin to the formation of intermolecular bonds [23].

Frequency shifts of the *HP-Fe* hydrogels peaks associated with –OH oscillations (3284→3274 cm^−1^) were observed towards smaller wave numbers in comparison with *H-Fe*. In general, this was an indication of the formation of a more bounded structure with tannin by the hydrogen bonds [24]. The peaks associated with deformation aromatic C-H (3073→3068 cm^−1^) and amide I (1633→1628 cm^−1^) were also displaced. The vibrations of the carbon bonds of the aliphatic structure CH_3_- (2922→2933 cm^−1^) and aromatic CH-OH (1402→1405 cm^−1^) shifted to a longer wavelength region. This also confirmed the proposed scheme of the formation of tannin bonds with other molecules. While the shift of the peak of amide I probably indicated the contribution of succinic acid residues to the fluctuations of the secondary amines associated with carbonyl groups.

### 2.6. Thermal Analysis

**Figure 7:**
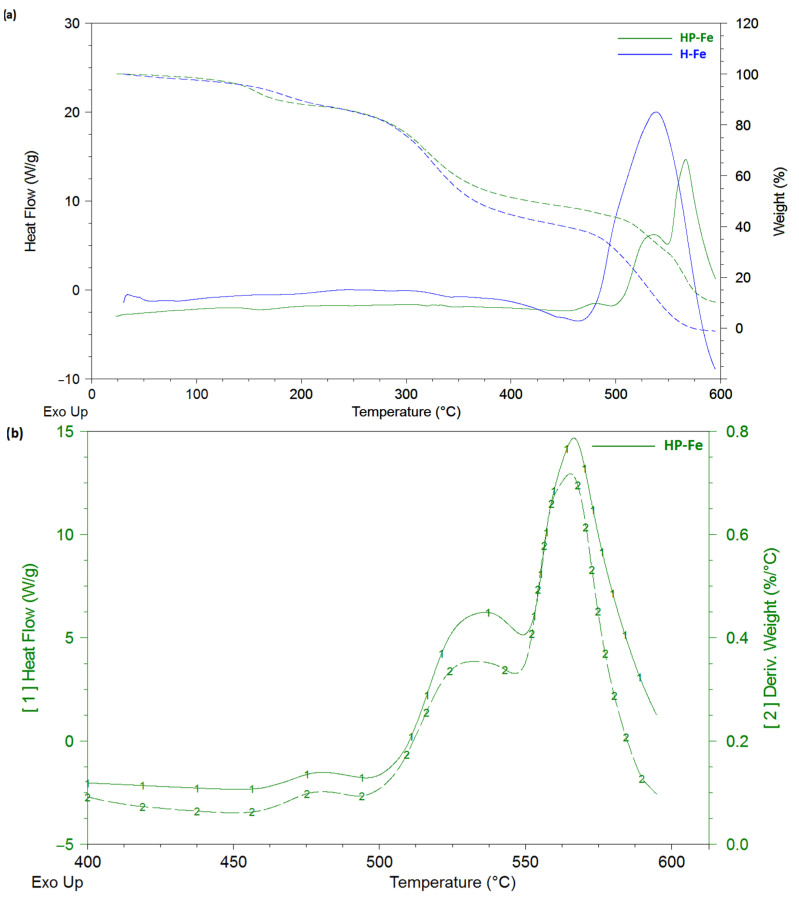
Thermal curves: (**a**) comparison of the thermal behavior of the *HP-Fe* and *H-Fe* hydrogels; (**b**) first heat and mass derivatives for *HP-Fe* hydrogels.

*HP-Fe* hydrogels showed a reduced stage of dehydration and cleavage of low-molecular-weight substances (up to 135 °C) in contrast to *H-Fe* (up to 150 °C). This may have been a sign of a more structured system, due to the preliminary binding of tannin by iron ions and gelatin by the succinate residues (Figure 7a). The consequence of which could have been a tendency toward a lower loss of water bounded by the polymer mesh [25]. The moisture content of the materials was almost the same (3.5 ± 0.1%). The stage of decomposition of the supramolecular structure (200–350 °C) also showed the identical character of the material thermal degradation.

The *Gelatinol*^®^-based hydrogels also showed an earlier onset of the third stage (decomposition of the structure), amounting to 350 °C compared to 375 °C in case of *H-Fe*. Three peaks are clearly distinguished in the heat flow curves, which were responsible for the three different stages of decomposition of the material. This is especially evident in the curves of the mass derivative (Figure 7b), in which *HP-Fe* has an almost identical character with the curves of the differential scanning calorimetry. It is also worth noting the much larger mass of the remainder at the end of the experiment (10.2% for *HP-Fe* and 1.5% for *H-Fe*). The higher temperature resistance also confirmed the success of the gelatin binding by the succinate residues.

### 2.7. Antimicrobial Activity

**Figure 8:**
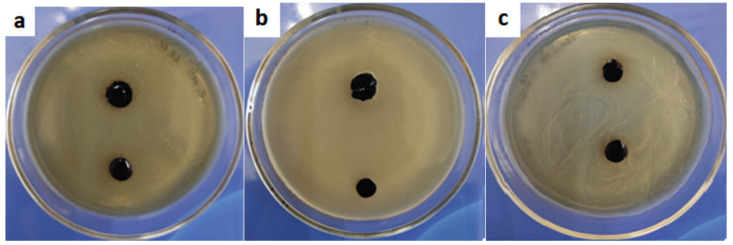
The state of the hydrogel 72 h after the antibacterial test with (**a**) *Escherichia coli*, (**b**) *Staphylococcus aureus*, and (**c**) *Candida albicans*.

In our study, the hydrogel film on the nutrient medium was resistant to dissolution; that is, it held its shape and borders, did not spread, and did not diffuse into the nutrient medium. We can also note that the material formed a stable antimicrobial coating for 24, 48, and 72 h for all types of studied microorganism (Figure 8).

## 3. Conclusions

This article revealed aspects of obtaining a highly adhesive resuscitation fluid-based hydrogel. Their prospective use lies in the field of wound dressing. IR spectroscopy and thermal analysis showed the successful formation of a crosslinked polymer structure. This highlighted the key features of the mesh and confirmed the proposed reaction scheme. The sorption parameters of the hydrogels at various physiological pHs were considered. The sorption kinetics demonstrated the pH-dependent behavior of the materials. The adhesion strength used to model skin reached 3.8 MPa in the shear test, demonstrating reliable adhesive properties. The hydrogels based on *Gelatinol*^®^ had a prevalent mixed nature of bond failure (70%), with less (30%) having a cohesive nature. The materials maintained a sufficiently high strength and elasticity of the hydrogels in the equilibrium swelled state. The permeability parameters were considered for oxygen and water steam, which showed the good transmittance of the coatings. In the future, synthesis technology can be further refined by usage of a catalyzing system. As most reactions represent nucleophilic substitution, it would probably be logical to assume possible phase-transfer catalyst usage. This would allow obtaining coatings in standard conditions, for further simplification of the production procedure. This would permit a larger sphere of application for the current materials. They may find a place in common medical, wound-healing, orthopedic, dental, and prothesis use. Moreover, it would be possible to use the proposed synthesis technology using 3D printing (by adjusting the viscosity with the help of fillers).

## 4. Materials and Methods

### 4.1. Materials

Reagents used: gelatin (type P-11, “TD-Holding”), tannin (food grade, “Lafayette”), NaOH (puriss., “Caustic”), FeCl_3_ (puriss., 6·H_2_O, “Reahim”), K_2_HPO_4_ (p.a., “Alphachem plus”), KH_2_PO_4_ (puriss., “Alphachem plus”), NaCl (puriss., “Tyretsky Solerudnik”), Na_2_HPO_4_ (p.a., 12·H_2_O, “Lenreactive”), KOH (p.a., “Nevareaktiv”) H_2_O_2_ 3% water solution (pharm., “Samara Pharmaceutical Factory”), H_3_PO_4_ (puriss. spec. type 12-3, “Nevareaktiv”, 1.71 g/cm^3^), citric acid (puriss., “Nevareaktiv”), succinic acid (p.a., “Nevareaktiv”), KI (puriss., “Eskay”), MnSO_4_ (pur., “Lenreactive”), Na_2_S_2_O_3_ (0.1n standard titr, TU 6-09-2540-72, “Ufa Chemical Company”), starch (p.a., “Lenreactive”), and ethanol (puriss.,“Nevareaktiv”). *Gelatinol*^®^ was obtained under a patent procedure [26]. Briefly, 8 g gelatin, 0.9 g potassium chloride, 0.2 g succinic anhydride, and 90.9 g water were mixed, sterilized, and neutralized to a pH about 6.7. As a result, a homogeneous yellow solution was obtained that did not undergo gelation up to +2 °C. Succinic anhydride was obtained by dehydration of succinic acid, the complete transformation of which was confirmed using IR spectroscopy and DSC.

### 4.2. Synthesis

**Figure 9:**
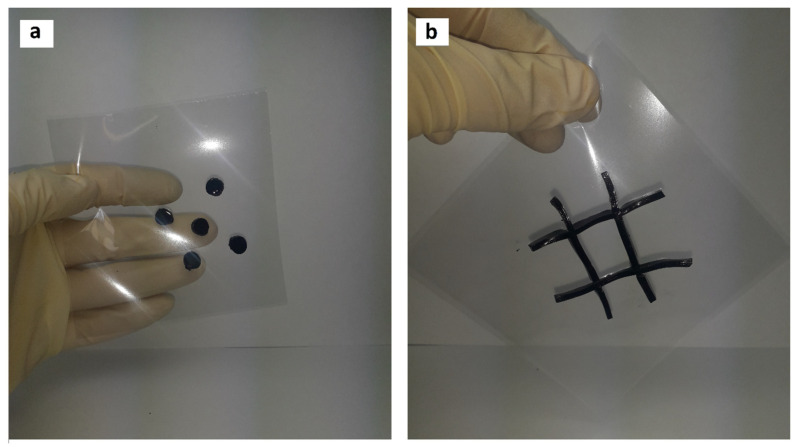
Synthesis of hydrogels of complex geometric shape, intended for the treatment of (**a**) chemical burns, (**b**) extensive burns.

First, 0.75 g tannin was added to 6.84 g hydrogen peroxide (3% mass.), and the pH was regulated to 10 (2M NaOH). The solution was blended and heated at 80 °C for one hour, followed by addition of 0.25 g ferric chloride. Then, 100 g of *Gelatinol*^®^ was added and mixed for 10 min at 70 °C. Next, the homogeneous solution was poured into petri dishes (Ø35 mm) or silicone molds. After pouring into molds and gelation (gelation characterization with DSC could be found in Supplementary Materials), the hydrogels were easily transferrable to a polyamide substrate, without violating the geometry of the material (Figure 9). Photos of the surface can be found in the Supplementary Materials.

As noted above, it is desirable to use these materials for specific types of skin damage, and not overlapping undamaged areas of the skin. In the current case, the production of gelatin–tannin hydrogels was demonstrated, which in the future could be used for the treatment of complicated [27,28] burns.

The hydrogels obtained using the described technology based on *Gelatinol*^®^ are hereinafter referred to as *HP-Fe*, and those obtained on the basis of a gelatin solution as *H-Fe*. Unless otherwise noted, samples in the form of disks with a diameter of 35 mm, a thickness of 0.1–0.15 mm, and a mass of 0.46 ± 0.02 g were subsequently examined. The samples were dried at 25 °C for 10 days, until the mass was constant (±0.01 g).

### 4.3. IR Spectroscopy

IR spectroscopy was performed on a Nicolet is −50 (ThermoFisherScientific, Lenexa, KS, USA) spectrometer with ATR accessory, supplied with ZnSe crystal (resolution −4 cm^−1^, scan number −16, wavenumber range −800–4000 cm^−1^, reflectance angle −45°, sample refractive index −1.4). The IR spectra were processed in Omnic v.1.11, which included correction of the baseline with the 2nd degree polynomial function, five-point smoothing, and spectrum normalization.

### 4.4. Thermal Analysis

Thermogravimetric analysis and differential scanning calorimetry were carried out on a thermal analyzer Q-600 (TA instruments) under the following conditions: weight of a sample −8 ± 0.5 mg, heating rate −10 °C/min, temperature range of the study (23–600) ± 1 °C, purge gas-air (40 mL/min), and open ceramic crucibles with a volume of 100 µL. Calibration of the device temperature range was carried out with indium (156.6 °C), tin (231.9 °C), and zinc (419.6 °C). The moisture content was defined as the ratio of the difference in the initial mass and mass loss at 130 °C to the initial mass. The gelation time was investigated in isothermal conditions under 25 °C for two hours, right after mixing. The curves were processed in TA Universal analysis 2000 v.4.5.

### 4.5. Mechanical Analysis

The tensile strength and elongation at break were determined on Instron 5966 (Instron), with the following parameters: the movement speed of the grippers was 10 mm/min, with rectangular samples with a size of 30 mm × 8 mm × 8 mm. The samples were cut from films (γ = 50 ± 5%) and placed with a distance of 10 mm between the grips. The adhesive strength was studied under the same conditions, the samples were 30-8 mm plates overlapped (10-10 mm) on top of each other: one of hydrogel, the other of chrome-tanned pigskin. The skin was degreased with ethyl alcohol. After the plates were placed, they were left until the hydrogel sample had dried to the required moisture content (defined by gravimetric method) and tested for the strength of the adhesive bond with the shear load.

### 4.6. Sorption

Phosphate buffer solutions (pH = 2–9) were prepared in accordance with the Russian Pharmacopeia (XIII ed.). The swelling of samples was investigated for 48 h, removing the samples at set time intervals, wiping the surface of excess water, and weighing. The degree of swelling was calculated according to Formula (1). The moisture content was defined from TGA data as the relative difference between initial mass and mass after isothermal heating (130 °C) for one hour.

Q_t_ = (m_t_ − m_o_(100 − γ))/(m_o_(100 − γ)) (1)

where m_o_ and m_t_, accordingly, are the mass of the sample at the beginning of the study and at time t, g; γ—moisture content, %.

The swelling kinetics study of the gelatin–tannin samples was carried out using the pseudo-second order equation and Fick’s first law, according to Formulas (2) and (3) [29]. Fick’s law describes diffusion only in the initial period, while the pseudo-second order equation describes the whole sorption process.

dQ_t_/dt = k_2_(Q_max_ − Q_t_)^2^ (2)

K·t^n^ = Q_t_/Q_max_ (3)

where Q_max_—swelling at the end of the experiment, g/g; t—time, min; k_2_—swelling velocity constant in equation of second pseudo order, min^−1^; K—swelling velocity constant in the Fick equation, g·(mmole·min)^−1^; n—degree of diffusion.

### 4.7. Water Vapor Permeability

The experiments were performed according to the water method of ASTM E96/E96M—10 [30] in a thermostat (CM 60-150/250-TVH, “SM Climat”, Russia) at temperature of 20 ± 1 °C and relative humidity 40 ± 5%. Polypropylene flasks were filled with distilled water to a mark of 5 ± 1 mm from the edge. Hydrogel films were placed on top of flasks with an effective area of 63.6 ± 0.5 cm^2^, fixing at the edges to prevent outside influence (not through the films). Two control samples without samples were used for comparison: a closed flask and a fully open one. Then, the sample containers were weighed and placed in a thermostat for 24 h. After that, they were weighed at certain time intervals and the vapor permeability was calculated using Formula (4):

WVTR = G/(t·A)(4)

where G/t—mass change with time, g/hour; A—effective area of a sample, m^2^.

### 4.8. Oxygen Permeability

First, 200 mL of water was poured into flat bottom flasks, the necks of which were covered with hydrogel films (area of the material 9.6 ± 0.3 cm^2^). Two control probes were used for comparison: opened (without hydrogel sample) and closed (with rubber plug). The flasks were placed in a thermostat under standard conditions for 24 h. The samples were removed from the necks and titrated (iodometric) to determine the content of active oxygen, according to a previously reported method [31].

### 4.9. Determination of Antimicrobial Activity

The antimicrobial properties of the samples were evaluated using the *Escherichia coli* (Gram-negative bacteria), *Staphylococcus aureus* (Gram-positive bacteria), and *Candida albicans* (yeast-like fungus) microbial strains. The experiments to evaluate the antibacterial properties were performed in accordance with the recommendations of the Clinical and Laboratory Standards Institute (CLSI) [32] and according to the European Committee on Antimicrobial Susceptibility Testing (EUCAST) [33]. Givental–Witch-type nutrient medium (AGV) was sterilized by autoclaving (121 ± 2 °C, 103 ± 5 kPa) for 20 min. Warm medium (20 mL) was poured into Petri dishes and allowed to cool at ambient temperature. The inocula for MIC were prepared in a sterile sodium chloride solution. Using a sterile Drigalsky spatula, the inoculate was evenly distributed over the entire surface of the agar with lawn seeding. For the disk diffusion method, the hydrogel material was cut in the form of disks with a diameter of 4 mm and placed on top of the inoculate seeding. The microorganisms were grown at 37 °C for 24, 48, and 72 h. The experiments were repeated three times.

### 4.10. Statistical Analysis

All data are presented in the format value ± standard deviation. The results were processed using a single-factor analysis of variance, with a statistical significance level (*p*) < 0.05 for each sample. Analysis was performed using Microsoft Excel 2016. The sample size for each test for mechanical tests—10 samples, and for sorption and permeability—6.

### 4.11. Other Measurements

The thickness of hydrogel films was measured with a micrometer MDC-1 MJC 293–330 (Mitutoyo). Linear dimensions were obtained using a caliper ShZ-I 0–150 (ChIZ). Analytical scales were used to determine the mass VL-120M (Gosmetr), discreteness—10^−5^ g. A MBS-9 (LOMO) microscope was used for the morphological investigation.

## Figures and Tables

**Table 1:** Sorption parameters of *HP-Fe*.

pH	*k*, min^−1^	*n*	*k*_2_·10^6^, g·(mmole·min)^−1^
2	4.50 ± 0.11	0.70 ± 0.03	3.14 ± 0.11
2.5	3.21 ± 0.09	0.53 ± 0.03	0.37 ± 0.02
3	4.17 ± 0.10	0.66 ± 0.02	1.30 ± 0.04
3.5	3.67 ± 0.09	0.61 ± 0.02	1.15 ± 0.04
4	2.77 ± 0.07	0.53 ± 0.01	0.22 ± 0.02
4.5	2.98 ± 0.08	0.53 ± 0.02	0.45 ± 0.02
5	2.85 ± 0.07	0.52 ± 0.02	0.43 ± 0.02
5.5	3.26 ± 0.09	0.55 ± 0.02	0.32 ± 0.02
6	1.19 ± 0.03	0.17 ± 0.01	0.49 ± 0.03
6.5	1.99 ± 0.04	0.35 ± 0.01	1.14 ± 0.04
7	1.23 ± 0.03	0.19 ± 0.01	0.42 ± 0.02
7.5	0.43 ± 0.01	0.05 ± 0.01	1.13 ± 0.04
8	4.35 ± 0.11	0.67 ± 0.03	0.34 ± 0.02
8.5	4.17 ± 0.10	0.64 ± 0.03	0.52 ± 0.03
9	3.98 ± 0.10	0.60 ± 0.02	0.94 ± 0.04

**Table 2:** Material permeability.

Sample	WWTR, g/m^2^	OP, mg/L
Open (control probe)	4510 ± 50	10.5 ± 0.03
*HP-Fe*	3610 ± 40	9.7 ± 0.02
Closed (control probe)	70 ± 5	9.1 ± 0.02

## Data Availability

The data presented in this study are available in the article.

## Supplementary Materials

The following supporting information can be downloaded at: https://www.mdpi.com/article/10.3390/gels9040330/s1, Figure S1—Adhesion test (samples No 5, 6, and 10 had cohesive failure, while the others had a mixed nature of bond destruction). Figure S2—Microphotos of *HP-Fe* surface. Figure S3—DSC thermogram of *HP-Fe* gelation. Figure S4—Conversion degree of gelation. Formula (S1)—Conversion degree.
