# Supplementary material for: Nanoarchitectonics of a Skin-Adhesive Hydrogel Based on the Gelatin Resuscitation Fluid Gelatinol®
Source: Gels. 2023 Apr 13;9(4):330. doi: 10.3390/gels9040330 (PMC10137424; doi:10.3390/gels9040330)
Supplement: Supplementary file 1 [file gels-09-00330-s001.zip › gels-2294078-supplementary.pdf]

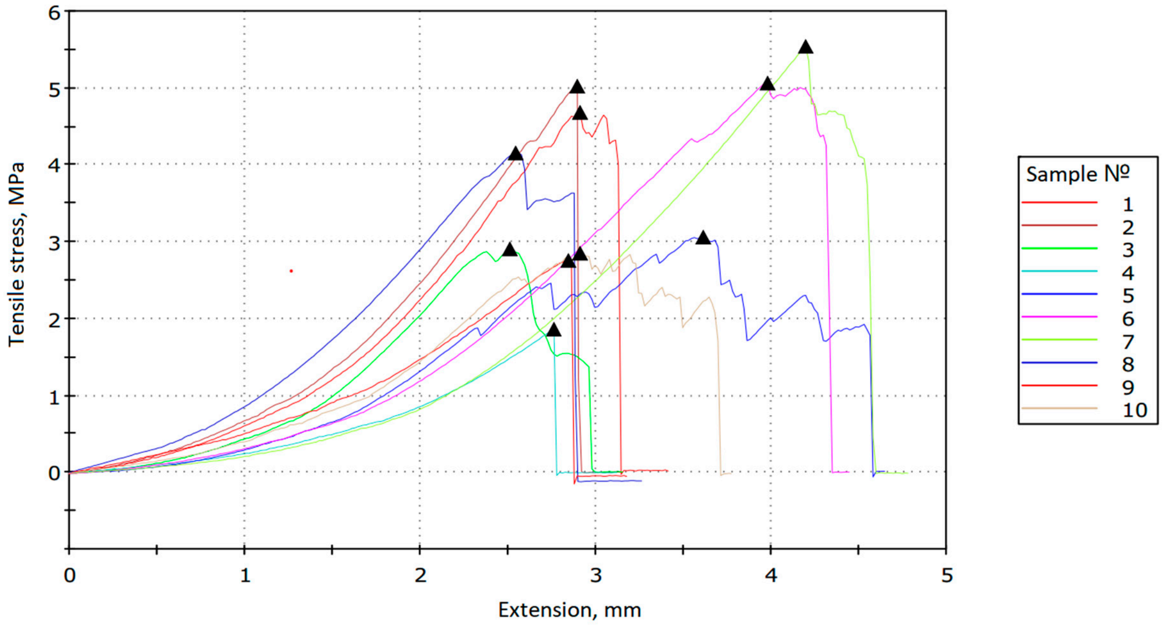

Figure S1 – Adhesion test (samples № 5, 6 and 10 had cohesive failure, while others had mixed nature of bond destruction).

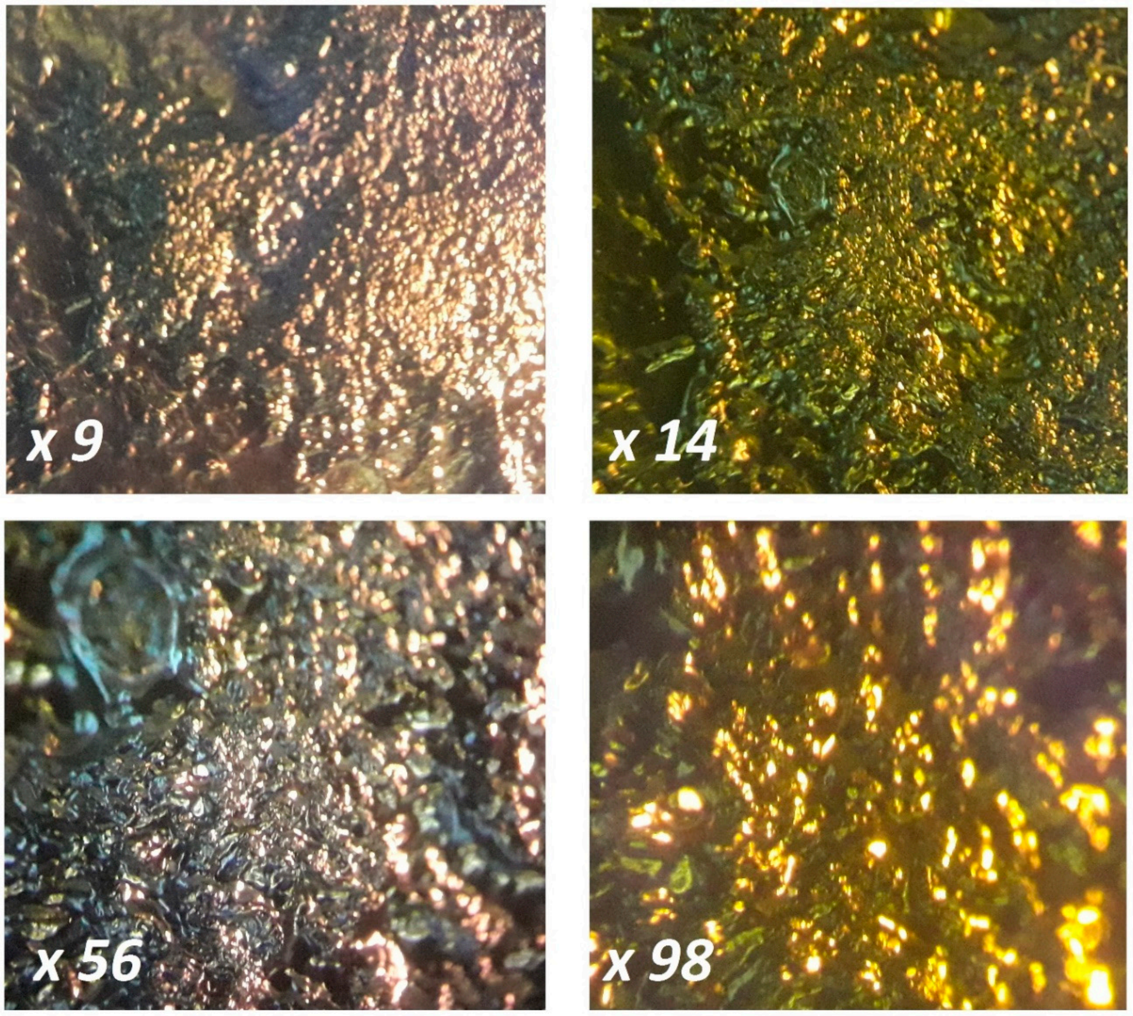

Figure S2 – Microphotos of HP-Fe surface.

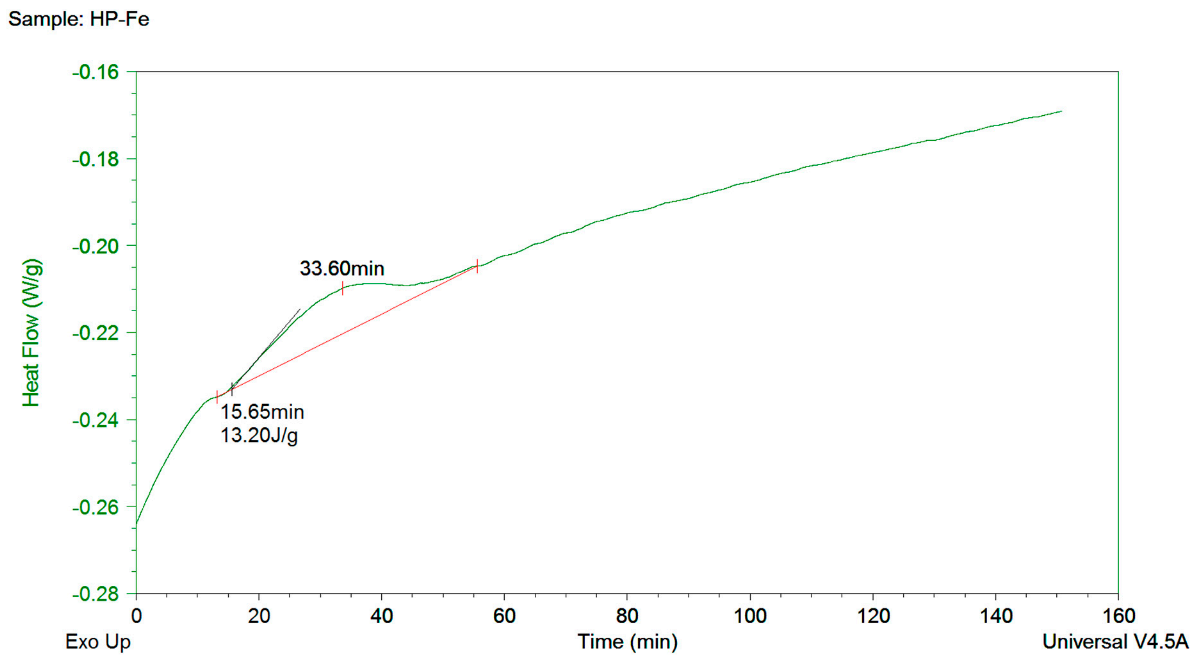

Figure S3 – DSC thermogram of HP-Fe gelation.

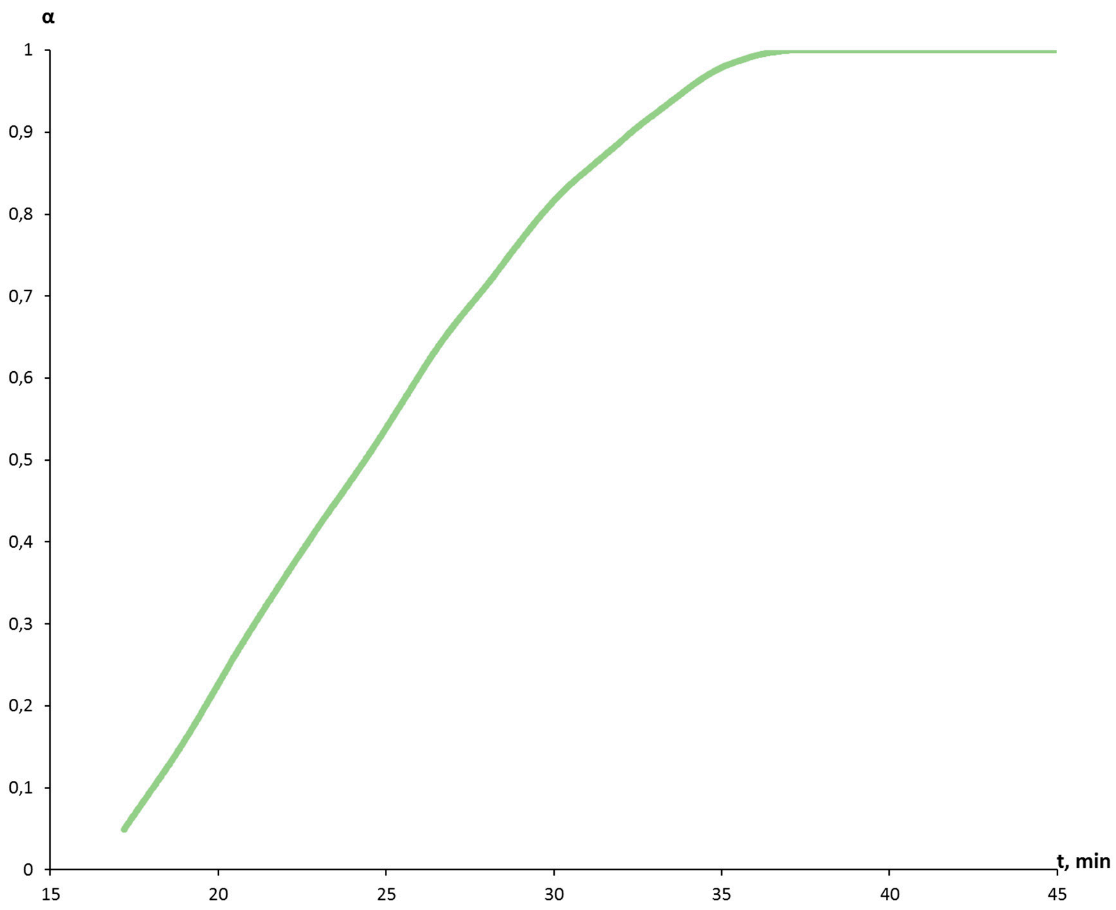

Figure S4 – Conversion degree of gelation.

$\alpha = \Delta H / \Delta H_t$ (S.1)

where ΔH *– enthalpy change at certain time, J/g,* ΔH_t *– total enthalpy of a gelation, J/g.*
